# Developmental Relations Between Internalising Problems and ADHD in Childhood: a Symptom Level Perspective

**DOI:** 10.1007/s10802-021-00856-3

**Published:** 2021-08-07

**Authors:** Lydia Gabriela Speyer, Manuel Eisner, Denis Ribeaud, Michelle Luciano, Bonnie Auyeung, Aja Louise Murray

**Affiliations:** 1 Department of Psychology, Univsersity of Edinburgh, Edinburgh, UK; 2 Violence Research Centre, Institute of Criminology, University of Cambridge, Cambridge, UK; 3 Jacobs Center for Productive Youth Development, University of Zurich, Zurich, Switzerland; 4 Autism Research Centre, Department of Psychiatry, University of Cambridge, Cambridge, UK

**Keywords:** ADHD, Internalising problems, Longitudinal network modelling, Z-proso

## Abstract

ADHD and internalising problems commonly co-occur with up to 50% of children diagnosed with ADHD also suffering from anxiety or depression. However, their developmental relations are currently not well understood. Longitudinal symptom level analyses can provide valuable insights into how difficulties in these areas of psychosocial functioning affect each other. Using Gaussian Graphical Models and Graphical Vector Autoregression Models, this study estimated cross-sectional and longitudinal networks of ADHD and internalising symptoms in 1387 children using parent- and teacher-reported Social Behaviour Questionnaires (SBQ) when children were aged 7, 9 and 11. Cross-sectional and longitudinal networks suggested that ADHD shares reciprocal relations with internalising symptoms through a number of potential bridge symptoms that are primarily connected to anxiety symptoms. High scores on *child cannot sit still, is restless, or hyperactive* were found to be the strongest bridge symptom acting as an antecedent to higher internalising symptoms whereas *child is worried* was the strongest antecedent for higher ADHD symptoms. Findings of this study highlight several potential bridge symptoms that may serve as key intervention targets and further emphasise the need for clinicians to assess children presenting with ADHD symptoms for internalising problems and vice versa.

**Supplementary Information:**

The online version contains supplementary material available at 10.1007/s10802-021-00856-3.

Attention Deficit Hyperactivity Disorder (ADHD) is one of the most common mental health issues in children, affecting around 6.5% of youths worldwide (Polanczyk et al., 2015). ADHD frequently co-occurs with internalising problems, with prevalence estimates in children ranging from 12 to 50% for co-occurring depression and from 15 to 35% for co-occurring anxiety (Gnanavel et al., 2019). However, relatively few studies have examined the links between ADHD symptoms and internalising problems longitudinally, making it difficult to draw any conclusions on the direction of their relations (Jarrett & Ollendick, 2008). In addition, most studies investigating the co-occurrence of ADHD and internalising problems have focused on clinical samples (Jarrett & Ollendick, 2008). Since both ADHD and internalising symptoms have been shown to lie on a continuum within the general population (Lubke et al., 2009; Tebeka et al., 2018), these studies should be complemented with community-based samples to provide a comprehensive picture of the links between ADHD and internalising problems.

Existing evidence on the longitudinal development of internalising problems and ADHD in normative samples suggests that they share reciprocal relations, with, for example, internalising problems leading to higher ADHD symptomatology and vice versa across mid- to late- adolescence (Murray et al., 2020a, b). Less is known about their relations before this period, which, in the context of the co-occurrence of ADHD and internalising symptoms, may be a particularly vulnerable period given that the median age-of-onset of internalising problems has been found to be at around age 11 (Kessler et al., 2005). ADHD typically manifests before the age of 7 but often only gets diagnosed after school entry as difficulties become more apparent in this setting (Sax & Kautz, 2003). Hence, to understand the etiology of co-occurring ADHD and internalising problems, it is of real importance to understand the temporal and concurrent relations of ADHD and internalising symptoms in the period leading up to adolescence.

To date, the mechanisms that underlie the relations between ADHD and internalising symptoms are still not well understood. This is also partly due to limitations in the methodology for analysing longitudinal panel data. Most longitudinal studies investigating the links between ADHD and internalising problems (e.g. Obsuth et al., 2020) have used modelling techniques such as cross-lagged panel models (CLPM), which conflate between- and within-person effects and thus provide ambiguous results regarding the development of co-occurring mental health issues (Berry & Willoughby, 2017). An alternative to traditionally used statistical techniques such as CLPMs are graphical vector autoregression (GVAR) models (Epskamp, 2020). GVAR models allow for the separation of within- and between-person effects and are consequently well suited to investigate dynamic relations between multiple mental health domains. GVAR models further have the advantage that they allow for an intuitive visualisation and interpretation of complex results, making it possible to not only investigate the relations between multiple mental health domains at the domain level but also at the symptom level.

Another factor that has thus far limited our understanding of the links between ADHD symptoms and internalising problems has been the focus on the disorder level rather than the symptom level. There has been some evidence that specific symptoms of internalising problems might be more relevant in their association with ADHD than others and vice versa (Michelini et al., 2015). In particular, symptoms of anxiety, such as excessive worrying, have been hypothesised to put additional drain on attentional resources, leading to inattentive behaviour (Zainal & Newman, 2020). On the other hand, attention problems might make it more difficult to shift attention away from ruminative thoughts and thus exacerbate internalising symptoms (Mitchell et al., 2013). Thus, some symptoms might be more important in the relations between ADHD and internalising problems, making them priority intervention targets. To identify these symptoms, symptom level analyses are needed. Indeed, symptom level analyses have recently gained in popularity due to a shift in the understanding of mental health disorders that has resulted in the development of the network approach to psychopathology (Borsboom, 2008). Rather than conceptualising mental health disorders as a collection of symptoms caused by a unitary underlying abnormality, the network approach to psychopathology views mental health disorders as dynamic networks of multiple mutually reinforcing symptoms with taxonomic classifications only serving as a means to describe a specific symptom network. This approach also allows for a more parsimonious understanding of co-occurring mental health problems given that symptoms between mental health disorders show substantial overlap (e.g. concentration difficulties are not only a symptom of ADHD but also of depression) (American Psychiatric Association, 2013). Hence, symptom networks between different disorders are likely to be connected through specific symptoms. These connecting symptoms are commonly referred to as ‘bridge symptoms’ and might act as the driving force in the development of a co-occurring disorder by activating another symptom network (Borsboom & Cramer, 2013). This has important implications for clinical interventions. Knowing which symptoms might underlie the development of another disorder allows for more targeted and consequently more effective interventions. To further maximise the impact of potential interventions, it is further crucial to know how these symptom networks change over development. In response to the emergence of the network approach to psychopathology, a number of studies have estimated cross-sectional symptom level networks (e.g. Beard et al., 2016; Rouquette et al., 2018; Silk et al., 2019), some also examining how these symptom networks changed over time (e.g. Martel et al., 2016), and providing insights into the structure of symptoms underlying mental health. However, while cross-sectional symptom networks can highlight potential bridge symptoms, they are limited in that they do not give any information on direction of effects. Understanding the direction of effects is critical as this could inform the etiology of psychopathology and enables interventions to target the right symptoms at the appropriate time. To date, there have been very few attempts to model the temporal relations between symptom networks, with the few studies attempting to model them longitudinally suffering from similar limitations as domain level analyses. Funkhouser et al. (2020), for instance, used cross‐lagged panel network analysis to analyse symptoms of internalising, externalising and attention symptoms over two time points, which like CLPMs, conflates within- and between-person effects. Thus, there is a clear need for more appropriate modelling of longitudinal symptom networks.

An additional limitation of many studies on the developmental relations of different mental health issues stems from the fact that they often rely on a single informant to measure children’s socio-emotional functioning. Evidence from cross-informant studies indicates that different informants only show small-to-moderate degrees of convergence in their assessment of children’s mental health, especially when these informants experience children in different contexts (e.g. home or school) (Murray et al., 2007, 2018). Consequently, studies investigating children’s mental health should replicate their findings based on assessments from at least one other informant. This is particularly relevant in the study of ADHD as, according to the DSM-5 diagnostic criteria, a diagnosis is only warranted if individuals show difficulties in at least two different settings (American Psychiatric Association, 2013).

In the current study, we investigate the developmental relations of internalising symptoms and ADHD symptoms in a large community-based study of N = 1387 children. Symptoms of anxiety, depression, inattention and hyperactivity/impulsivity were measured at median-ages 7, 9 and 11 using parent-reported Social Behaviour Questionnaires (SBQ). Using Gaussian Graphical Models (GGM) and Graphical Vector Autoregression Models, we estimate cross-sectional as well as longitudinal symptom networks of ADHD and internalising problems to gain new insights into concurrent and temporal relations of symptoms bridging ADHD and internalising problems. To evaluate the stability of our results across informants, we further replicate all cross-sectional models using teacher-reported SBQs. As this is the first study to investigate the symptom-level relations of ADHD symptom and internalising problems longitudinally, we took an exploratory approach.

## Methods

### Participants

Participants in this study were part of the Zurich Project on Social Development from Childhood to Adulthood (z-proso), a longitudinal study based in Zurich, Switzerland, that has been tracking the development of an initial target sample of 1675 children from 2004 when the children entered school at age 7. Children were recruited based on a stratified sampling design whereby 56 public primary schools in Zurich were selected based on school size and location. Participants were ethnically diverse with only 39.6% of primary caregivers being native speakers of the official language of Zurich, that is German, after which the most frequently spoken native languages were Serbian/Bosnian/Croatian (10%), Albanian (9%), Portuguese (7%) and Tamil (5.3%) (Eisner et al., 2019). To maximize engagement of the non-German-native speakers, contact letters as well as parent interviews were made available in the in the ten most commonly spoken languages. Of the children’s male primary caregivers who contributed to the first wave of data collection, 76.7% were in full-time employment (8.8% unemployed), with 16% having a university-level education, 15.5% a higher vocational education 7.8% A-levels, 35.2% apprenticeship and 21% mandatory school or less (Murray et al., 2016). To date, there have been ten waves of data collection at ages 7 to 13, 15, 17 and most recently at age 20 with data collection still ongoing. This study uses data from waves at median-ages 7, 9 and 11 at which the same items on internalising and ADHD symptoms were available from parents and teachers. At the age 7 wave, data was available for 1370 youths, at the age 9 wave for 1321 and at the age 11 wave for 1147 youths. All children who had data on internalising symptoms and ADHD for at least one time-point from at least one informant were included in the current study, resulting in a final sample of 1387 children (51% male).

The z-proso study obtained ethical approval from the Ethics Committee from the Faculty of Arts and Social Sciences of the University of Zurich. Parents provided active informed consent for children to participate in the study. For additional details regarding recruitment, retention, and attrition, see elsewhere (Eisner & Ribeaud, 2007; Eisner et al., 2019) and the z-proso website (https://www.jacobscenter.uzh.ch/en/research/zproso/aboutus.html).

### Measures

Symptoms of ADHD and internalising problems were measured using parent- and teacher-reported versions of the Social Behaviour Questionnaire (SBQ; Tremblay et al., 1991). The SBQ is an omnibus measure of psychopathology and measures children’s psychosocial functioning across five areas: ADHD, anxiety/depression, aggression, non-aggressive externalizing problems, and prosocial behaviour. At ages 7, 9 and 11 in the z-proso study, SBQs were completed by parents and teachers and included four items each on symptoms of inattention, hyperactivity/impulsivity, and depression, as well as three items on symptoms of anxiety. Items were rated on a 5-point Likert scale from *Never* to *Very Often.* SBQs were administered using a German translation of the original SBQ which teachers completed in the form of a paper-and-pencil questionnaire while parents took part in a computer-assisted personal interview that was available in an additional nine languages for non-German speaking participants. For English phrasings of the items used in this study see Table 1. While self-reported SBQs were also available, those were not used in the current study as they were collected in the form of an adapted computer-based multimedia version of the SBQ with children answering ‘yes’ or ‘no’ to a series of questions relating to their psycho-social development. Psychometric analyses of the SBQ have found support for factorial validity, developmental invariance and criterion validity of the SBQ items across various waves of the z-proso study (Murray et al., 2017, 2020a, b). The SBQ has further been shown to be a reliable measure of moderately low to very high levels of internalising and ADHD symptoms in the general population (Murray et al., 2019). Descriptive Statistics as well as correlation tables of all SBQ items included in the current study are available online (Table S1 in the supplementary and excel files E1 and E2).

**Table 1:** ADHD and Internalising Problems Items

Item	Domain	Item Content
02 (NER)	Anxiety	< CHILD > is nervous, high-strung or tense
03 (ANX)	Anxiety	< CHILD > is too fearful or anxious
04 (WOR)	Anxiety	< CHILD > is worried
05 (DEP)	Depression	< CHILD > seems to be unhappy, sad, or depressed
06 (NHA)	Depression	< CHILD > is not as happy as other children
07 (TEN)	Depression	< CHILD > has trouble enjoying him\herself
08 (DIS)	Depression	< CHILD > appears miserable, distressed, or unhappy
10 (IMP)	Hyperactivity/impulsivity	< CHILD > is impulsive, acts without thinking
11 (DWA)	Hyperactivity/impulsivity	< CHILD > has difficulty awaiting turn in games or groups
12 (RES)	Hyperactivity/impulsivity	< CHILD > cannot sit still, is restless, or hyperactive
13 (FID)	Hyperactivity/impulsivity	< CHILD > fidgets
14 (CSE)	Inattention	< CHILD > cannot settle to anything for more than a few moments
15 (DTB)	Inattention	< CHILD > is distractible, has trouble sticking to any activity
16 (CON)	Inattention	< CHILD > cannot concentrate, cannot pay attention for long
17 (INA)	Inattention	< CHILD > is inattentive

### Statistical Analyses

To improve our understanding of the concurrent relations between symptoms of ADHD and internalising problems, a series of cross-sectional networks was estimated using Gaussian Graphical Models (GGM). For each time point (ages 7, 9 and 11), separate networks for parent- and teacher-reported symptoms were built. GGMs use partial correlations to intuitively visualise the complex dependence structures of a system of variables. In GGMs, variables are represented by nodes that are connected through directed (temporal network) or undirected (contemporaneous network) edges which visualise the relations between variables (Epskamp et al., 2018a, b). Edge weights (*w*) quantify the strength of the association in the form of partial correlations. Networks were estimated using the R package *qgraph* (Epskamp et al., 2012) which implements graphical LASSO (least absolute shrinkage and selection operator) regularization in combination with Extended Bayesian Information Criterion (EBIC) as model selection criterion to estimate a sparse network structure (Epskamp & Fried, 2018). Using the R-package *bootnet* (Epskamp et al., 2018a, b)*,* 95% Confidence Intervals (*CIs*) for edge weights were obtained through bootstrapping routines (N = 1000). Lastly, cross-sectional networks at different time points as well as parent- and teacher-reported models were compared using permutation based Network Comparison Tests (NCT) which offer information on whether networks differed in global network strength (*S:* sum of all edge weights; *DiffS*: Difference in *S* between two networks) and in network structure (*M*) (Van Borkulo et al., 2015).

To analyse the longitudinal relations between ADHD and internalising symptoms, a Graphical Vector Autoregression (GVAR) model was built for parent-reported data using the panelgvar() function of the R package *psychonetrics* (Epskamp, 2020). GVAR models describe variables that have been measured at several time points as a function of their own past values or as a combined function of their own as well as other variables’ past values, allowing insights into temporal as well as concurrent relations between several repeatedly measured variables (Wild et al., 2010). These temporal and concurrent relations can be visualised using GGMs that include directed edges to visualise temporal relations and undirected edges to visualise concurrent relations. If the data includes a multilevel structure, GVAR models also estimate cross-sectional between-person differences across time, which enables the separation of within- from between-person effects (Epskamp, 2020). This separation is critical since between-person effects, which describe how someone’s average on a specific symptom compares to someone else’s average on another symptom, act as a confound when the interest lies on investigating within-person effects. Within-person effects describe individuals’ deviations from their own average symptom levels and can give insights into whether high- or low-levels on one symptom influence that same person’s levels on another symptom. Investigating within-person effects is critical as they can give insights into the mechanisms that might underlie the associations between two symptoms and represent the prime target for interventions (Hamaker et al., 2015). Structurally, GVAR models are closely related to the Random-Intercept Cross-lagged Panel Model (RI-CLPM) which separates within- from between-person effects by partialling out stable-between person differences through random intercepts for each repeatedly measured variable that are allowed to co-vary (Hamaker et al., 2015). In contrast to RI-CLPMs which model the within- and between-person covariance structures as marginal variance–covariance matrices, GVAR models, however, model these as GGMs. Also, to avoid the need for estimating a variance–covariance structure for the first measurement point, GVAR models assume stationary relations, thus, unlike RI-CLPMs, they treat the first measurement wave as endogenous (Epskamp, 2020).

Before building the GVAR models, data was detrended for linear age-related effects and standardised across time points to meet the stationarity assumption of GVAR models. This was considered appropriate for the current analyses because only the correlational structure and not the mean structure was of interest. In order to appropriately account for missing data, the GVAR model was fitted using Full Information Maximum Likelihood (FIML) estimation which provides unbiased estimates under the assumption that data is missing at random (Enders, 2001). To minimise the chance of finding false positives and to reduce the model’s complexity, the model was further regularised (i.e., constrained to only include the most important edges) using Bayesian Information Criterion (BIC) as model selection criterion. Overall model fit was judged using the following relative fit indexes: Comparative Fit Index (CFI), Tucker Lewis Index (TLI) and Root Mean Square Error of Approximation (RMSEA) with CFI > 0.90, TLI > 0.90 and RMSEA < 0.05 used as cut off criteria indicating reasonably good fit (Kline, 2005). Due to computational limitation, bootstrapping routines could not be employed for the GVAR model. Unfortunately, replicating the parent-reported GVAR model using teacher-reported SBQs was also not possible as GVAR estimations failed due to numerical optimization issues likely caused by very high correlations (> 0.90) between some teacher-reported SBQ items. Given the computational complexity involved in fitting GVAR models, this is not unexpected.

All estimated networks were visualised using the Fruchterman-Reingold algorithm in the *qgraph* package which places nodes sharing stronger connections closer together (Epskamp et al., 2012). For cross-sectional networks, the network layout was kept constant (using average layout of all networks) to facilitate comparisons. To identify the most influential bridge symptoms, bridge influence indices were estimated using the R package *networktools*, giving insights into the direct and indirect influence of specific bridge symptoms on symptoms from the other area of psychosocial functioning (Jones et al., 2019).

## Results

### Cross-sectional Parent-Reported Networks

**Fig. 1:**
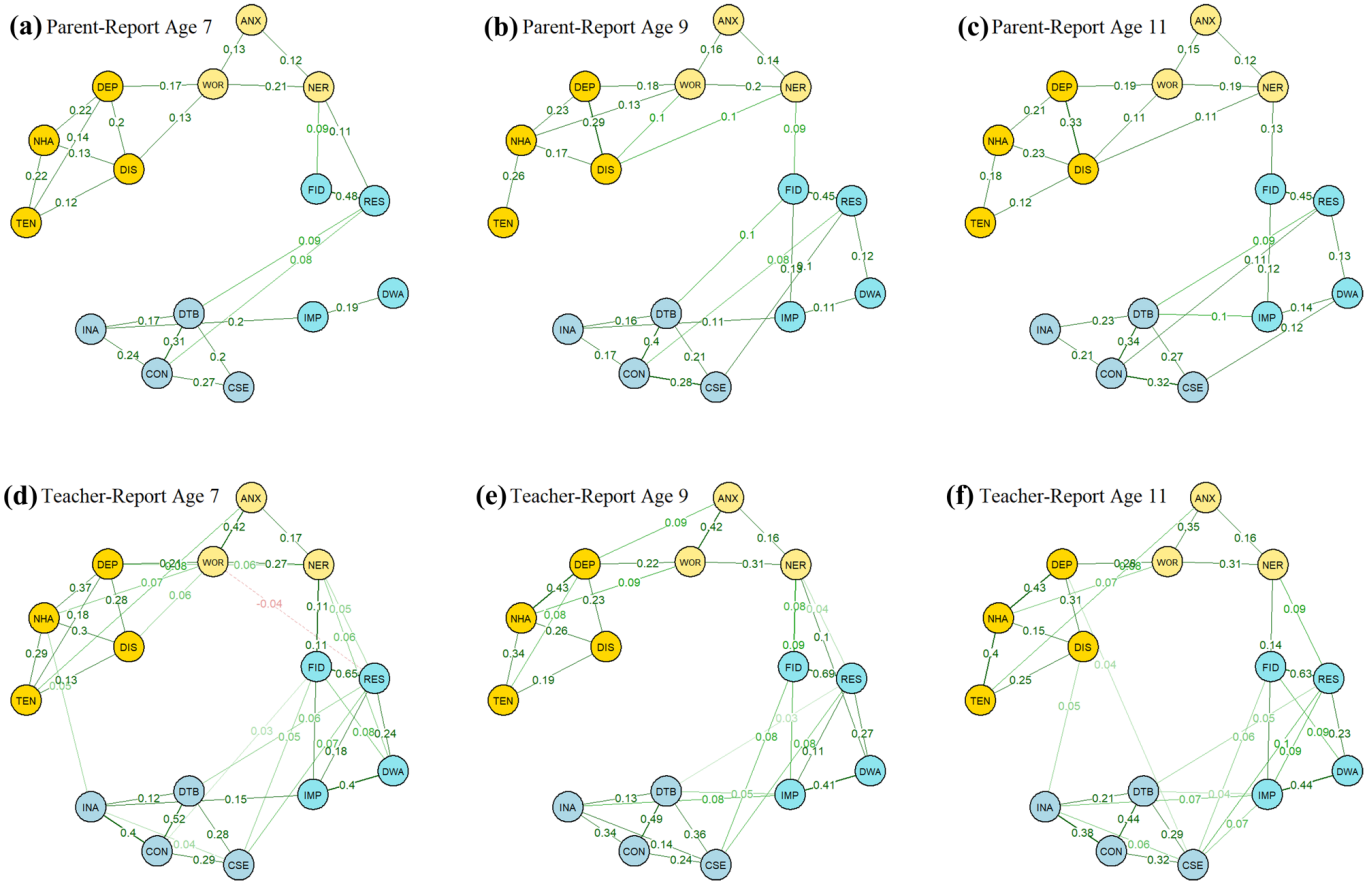
Cross-sectional partial correlation networks for parent- and teacher-reported symptoms at ages 7, 9, and 11. Green edges (solid lines) indicate positive effects; red edges (dashed) indicate negative effects. Upper row (**a**, **b**, **c**) represents parent-reported networks, lower row (**d**, **e**, **f**) represents teacher-reported networks. NER: *nervous*, ANX: *anxious*, WOR: *worried*; DEP: *depressed*, NHA: *not as happy as other children*, TEN: *trouble enjoying themselves*, DIS: *distressed*, IMP: *impulsive*, DWA: *difficulty awaiting turns*, RES: *restless*, FID: *fidgets*, CSE: *cannot settle to anything*, DTB: *distractible*, CON: *cannot concentrate*, INA: *inattentive*

All parent-reported cross-sectional networks showed clusters on the domain level with symptoms being more closely connected to other symptoms from their own domain than to symptoms from other domains (see Fig. 1). Internalising domains (i.e., anxiety and depression) and ADHD domains (i.e., hyperactivity/impulsivity and inattention) also formed distinct clusters. At every time point, these clusters were connected through links between item 13 (FID, *Child fidgets*) from the hyperactivity/impulsivity domain and item 2 (NER, *Child is nervous, high-strung or tense*) from the anxiety domain. At age 7, item 2 further shared an edge with item 12 (RES, *Child cannot sit still, is restless, or hyperactive*) from the hyperactivity/impulsivity domain. Bridge influence indices confirmed these visual findings (see Table 2). Pairwise NCTs indicated that network structures and global network strength (*S*_*7*_ = 5.97, *S*_*9*_ = 6.06, *S*_*11*_ = 6.25) were invariant over time with all comparisons yielding *p*-values larger than 0.05 (*M*_*7vs9*_ = 0.10. *M*_*7vs11*_ = 0.12, M_9vs11_ = 0.10; *DiffS*_*7vs9*_ = 0.10, *DiffS*_*7vs11*_ = 0.28, *DiffS*_*9vs11*_ = 0.19). Results of bootstrapping routines indicated that edges between bridge symptoms were moderately stable. Confidence intervals quantifying the uncertainty associated with all estimated edges are presented in the online supplementary Tables S2—S4.

**Table 2:** Bridge Influence Indices for Cross-Sectional Networks

	**Parent-Report: Age 7**		**Parent-Report: Age 9**		**Parent-Report: Age 11**	
Item	*Direct Influence*	*Indirect Influence*	*Direct Influence*	*Indirect Influence*	*Direct Influence*	*Indirect Influence*
02	0.21	0.33	0.09	0.15	0.13	0.20
03	-	0.03	-	0.01	-	0.02
04	-	0.04	-	0.02	-	0.03
05	-	-	-	-	-	-
06	-	-	-	-	-	-
07	-	-	-	-	-	-
08	-	-	-	0.01	-	0.01
10	-	-	-	0.01	-	0.02
11	-	-	-	-	-	-
12	0.11	0.20	-	0.04	-	0.06
13	0.09	0.18	0.09	0.13	0.13	0.18
14	-	-	-	-	-	-
15	-	0.01	-	0.01	-	-
16	-	0.01	-	-	-	-
17	-	-	-	-	-	-
	**Teacher-Report: Age 7**		**Teacher-Report: Age 9**		**Teacher-Report: Age 11**	
Item	*Direct Influence*	*Indirect Influence*	*Direct Influence*	*Indirect Influence*	*Direct Influence*	*Indirect Influence*
02	0.33	0.59	0.32	0.55	0.23	0.43
03	-	0.05	-	0.05	-	0.04
04	-.04	0.01	-	0.10	-	0.09
05	-	0.03	-	-	0.04	0.09
06	0.05	0.08	-	-	-	0.02
07	-	0.02	-	-	-	0.01
08	-	0.01	-	-	0.05	0.10
10	0.11	0.20	0.10	0.19	0.14	0.22
11	0.06	0.15	0.10	0.20	-	0.09
12	0.09	0.10	0.04	0.15	0.09	0.15
13	0.11	0.17	0.09	0.14	-	0.06
14	-	0.01	-	0.01	0.04	0.10
15	-	0.01	-	0.01	-	0.03
16	-	0.02	-	-	-	0.03
17	0.05	0.12	-	0.01	0.05	0.10

Bridge influence indices were derived from edge weights (partial correlations) in the respective cross-sectional network model. Direct influence: sum of all edge weights that exist between a node X and all nodes that are not part of the same cluster as node X (i.e. either ADHD or internalising). Indirect influence: direct influence plus indirect effects of Node X through other nodes (e.g. indirect effect on node Z as in X—Y—Z)

### Cross-Sectional Teacher-Reported Networks

Networks based on teacher-reports showed similar patterns to parent-reports with symptoms from the same domains forming distinct clusters (see Fig. 1). In line with the parent-reported networks, pairwise NCTs indicated that network structures and global network strength were invariant over time (*M*_*7vs9*_ = 0.10. *M*_*7vs11*_ = 0.15, M_9vs11_ = 0.12; *S*_*7vs9*_ = 0.19, *S*_*7vs11*_ = 0.24, *S*_*9vs11*_ = 0.05). With regards to bridge symptoms, teacher-reported networks also identified anxiety item 2 (NER, *Child is nervous, high-strung or tense*) and ADHD items 12 (RES, *Child cannot sit still, is restless, or hyperactive*) and 13 (FID, *Child fidgets*), as potential bridge symptoms. However, they further highlighted a number of additional connections bridging the internalising and ADHD domains. In particular, item 2 (NER, *Child is nervous, high-strung or tense*) was connected to all symptoms of the hyperactivity/impulsivity domain in the age 7 and age 9 networks, while only sharing edges with item 10 (IMP, *Child is impulsive, acts without thinking*) and 12 (RES, *Child cannot sit still, is restless, or hyperactive*) in the age 11 network. The age 7 network further highlighted a connection between the inattention and depression domains through item 17 (INA, *Child is inattentive*) and item 6 (NHA, *Child is not as happy as other children*). At age 11, item 17 was connected to item 8 (DIS, *Child appears miserable, distressed, or unhappy*) instead of item 6. In addition, item 14 (CSE, *Child cannot settle to anything for more than a few moments*) shared an edge with item 5 (DEP, *Child seems to be unhappy, sad, or depressed*). For bridge influence indices, see Table 2. The higher connectivity of the teacher-reported networks was also reflected in higher values for global network strength compared to parent-reported networks (*S*_*7*=_7.24, *S*_*9*_ = 7.43, *S*_*11*_ = 7.48). NCTs showed that these differences were significant at each time point (*p* < 0.001; *DiffS*_*7*_ = 1.27, *DiffS*_*9*_ = 1.36, *DiffS*_*11*_ = 1.22). Network structures were also found to be significantly different for parent- and teacher-reported networks (*p* < 0.001; *M*_*7*_ = 0.30. *M*_*9*_ = 0.34, *M*_11_ = 0.31). Similar to the parent-reported networks, estimates for edges between bridge symptoms based on teacher-reports were moderately stable. For confidence intervals, see Tables S2 – S4 in supplementary materials.

### Longitudinal Parent-Reported Network

**Fig. 2:**
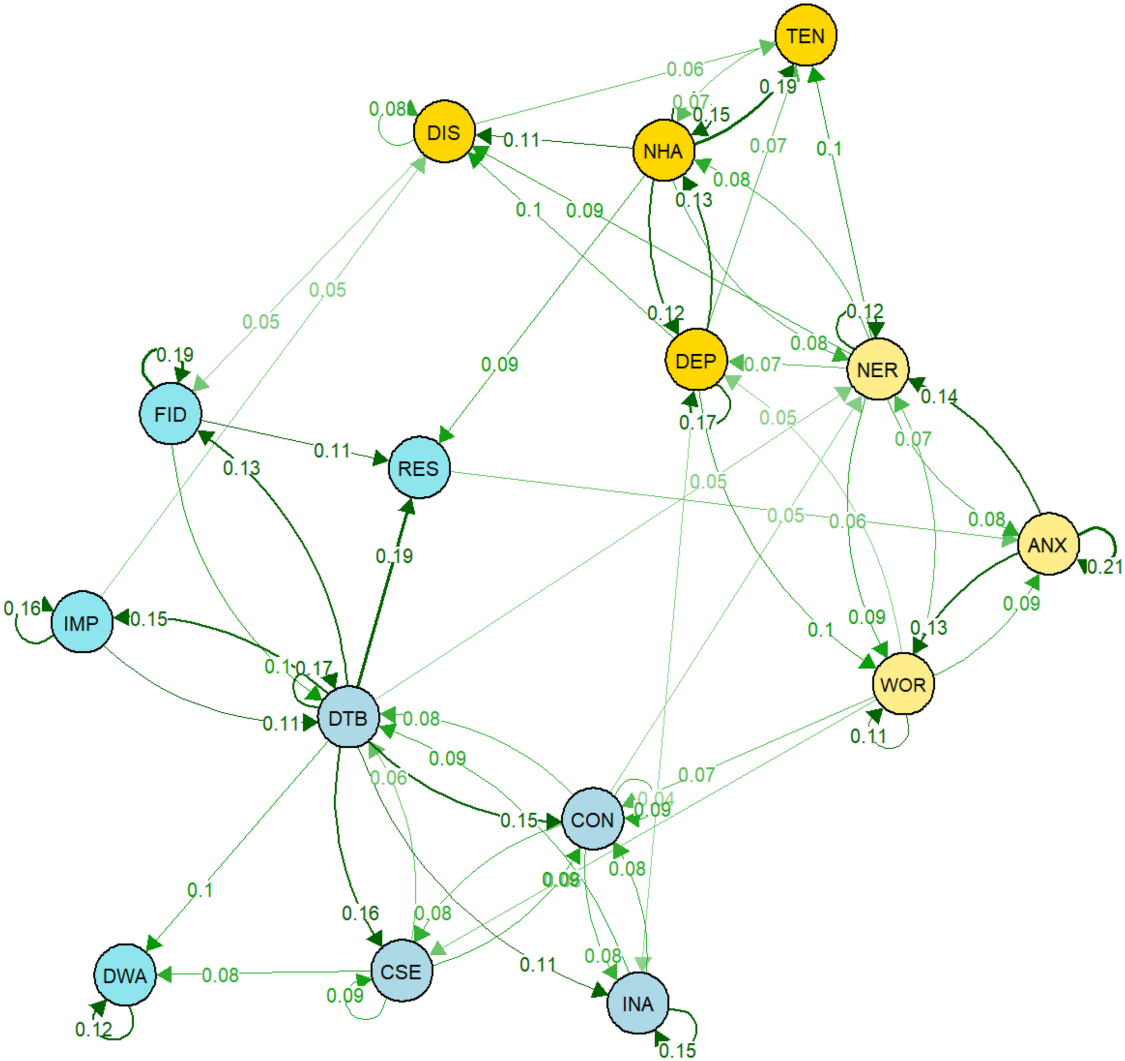
Temporal network for parent-reported symptoms standardised to directed partial correlations. Green edges (solid lines) indicate positive effects. NER: *nervous*, ANX: *anxious*, WOR: *worried*; DEP: *depressed*, NHA: *not as happy as other children*, TEN: *trouble enjoying themselves*, DIS: *distressed*, IMP: *impulsive*, DWA: *difficulty awaiting turns*, RES: *restless*, FID: *fidgets*, CSE: *cannot settle to anything*, DTB: *distractible*, CON: *cannot concentrate*, INA: *inattentive*

The saturated parent-reported GVAR model showed good fit (*CFI* = 0.96, *TLI* = 0.95, *RMSEA* = 0.028 with 90% *CI*: 0.026 to 0.030). The regularised model performed slightly better than the saturated model (*∆BIC* = 1696.74; *CFI* = 0.94, *TLI* = 0.93, *RMSEA* = 0.032 with 90% *CI*: 0.030 to 0.034). Overall, the temporal network indicated that, at the within-person level, most symptoms affected other symptoms over time, though symptoms shared more and stronger connections with symptoms from the same domain. With regards to edges bridging ADHD and internalising domains, hyperactivity/impulsivity items 10 (IMP, *Child is impulsive, acts without thinking*) and 12 (RES, *Child cannot sit still, is restless, or hyperactive*) had positive temporal effects on internalising symptoms from the depression and anxiety domains respectively, while items 15 (DTB, *Child is distractible, has trouble sticking to any activity*) and 16 (CON, *Child cannot concentrate, cannot pay attention for long*) from the inattentive domain were associated with increased anxiety symptoms. Regarding the effects of internalising on ADHD symptoms, item 4 (WOR, *Child is worried*) from the anxiety domain had positive temporal effects on inattentive ADHD symptoms and items 6 (NHA, *Child is not as happy as other children*), and 8 (DIS, *Child appears miserable, distressed, or unhappy*) from the depression domain were associated with increased hyperactivity/impulsivity ADHD symptoms. Interestingly, except for one direct link between ADHD symptom 10 (IMP, *Child is impulsive, acts without thinking*) and depression item 8 (DIS, *Child appears miserable, distressed, or unhappy*), ADHD symptoms mostly had positive directional effects on anxiety items. These in turn shared directional links with depression items, indicating that anxiety symptoms potentially mediate the relations between ADHD and depression symptoms. Out of all symptoms, bridge influence indices (Table 3) indicated that ADHD item 12 (RES, *Child cannot sit still, is restless, or hyperactive*) from the hyperactivity/impulsivity domain had the strongest direct influence on internalising symptoms, mainly from the anxiety domain, and internalising item 4 (WOR, *Child is worried*) from the anxiety domain had the strongest direct influence on ADHD symptoms, mainly from the inattention domain. The temporal within-person network is visualised in Fig. 2. The contemporaneous network, which is visualised in Figure S1 in the online supplementary, indicated that anxiety item 4 (WOR, *Child is worried)* shared particularly many relations with items from both ADHD domains, thus, suggesting that at the within-person levels, children with higher symptoms of worrying also tend to have higher ADHD symptoms at the same time-point. The between-person network (visualised in Figure S2, available online) indicated that children who have high scores on anxiety item 2 (NER, *Child is nervous, high-strung or tense*), tended to also have higher scores on hyperactive/impulsive ADHD symptoms compared to children who had lower scores on item 2 (NER, *Child is nervous, high-strung or tense*). Overall, the between-person network showed relatively little similarity with the within-person contemporaneous and temporal network, highlighting the necessity of appropriately disentangling within- from between-person effects when within-person effects are of primary interest as in the context of mental health interventions.

**Table 3:** Bridge Influence Indices for the Parent-Reported Temporal GVAR Network

Item	*Out-Strength*	*In-Strength*	*Direct Influence*	*Indirect Influence*
02	-	0.10	-	0.03
03	-	0.06	-	0.02
04	0.13	-	0.13	0.17
05	0.04	-	0.04	0.09
06	0.09	-	0.09	0.12
07	-	-	-	0.01
08	0.06	0.05	0.06	0.07
10	0.06	-	0.05	0.07
11	-	-	-	-
12	0.06	0.09	0.06	0.08
13	-	0.06	-	0.02
14	-	0.06	-	0.01
15	0.05	-	0.05	0.11
16	0.05	0.07	0.05	0.08
17	-	0.04	-	0.01

Bridge influence indices were derived from edge weights (partial correlations) in the temporal GVAR model. Out-Strength: Sum of the absolute values of out-degree edge weights (i.e. edges with a directional arrow from node X to another node). In-Strength: Sum of the absolute edge weights of in-degree edges (i.e. edges with a directional arrow from another node to node X). Direct influence: sum of all edge weights that exist between a node X and all nodes that are not part of the same cluster as node X (i.e. either ADHD or internalising). Indirect influence: direct influence plus indirect effects of Node X through other nodes (e.g. indirect effect on node Z as in X—> Y—> Z). Since, these networks are directed, influence measures only include out-degree edges

## Discussion

In this study, we used graphical vector autoregression models to investigate the development of ADHD and internalising symptom networks over time. This approach provides an important advance over cross-sectional symptom level networks as it allows for the estimation of directional relations that can improve our understanding of the development of psychopathology and inform interventions. Our results highlighted a number of potential bridge symptoms between these areas of psychosocial functioning. On the cross-sectional level, ADHD and internalising symptoms were primarily connected through item ‘*Child cannot sit still, is restless, or hyperactive*’ and item ‘*Child fidgets*’ from the hyperactivity/inattention domain and ‘*Child is nervous, high-strung or tense*’ from the anxiety domain. On the temporal level, anxiety item ‘*Child is worried*’ was the strongest direct antecedent of higher ADHD symptoms and item ‘*Child cannot sit still, is restless, or hyperactive*’ was the strongest direct antecedent of higher internalising symptoms.

In line with previous studies using cross-sectional symptom networks (Beard et al., 2016; Silk et al., 2019), our results indicated that items from the same domain (i.e. anxiety, depression, hyperactivity/impulsivity, inattention) formed relatively distinct clusters with internalising domains and ADHD domains clustering more closely together. Nevertheless, all cross-sectional networks highlighted that these internalising and ADHD symptom clusters are connected to each other through items from the anxiety and the hyperactivity/impulsivity domain, supporting the conceptualisation of co-occurring psychopathology as connected networks of symptoms. In particular, ADHD symptoms formed a bridge to internalising symptoms through an association with how often a child was nervous. Higher scores on items relating to restlessness and fidgeting were associated with higher scores on the nervousness item. Nervousness often goes hand in hand with restlessness, making it a plausible bridge symptom between anxiety and ADHD. Also, the fact that restlessness was part of the bridge to internalising problems is in line with DSM-5 diagnostic criteria which lists restlessness as a symptom of both ADHD and anxiety disorders (American Psychiatric Association, 2013).

Whereas cross-sectional models revealed bridges between symptom networks of ADHD and internalising problems, longitudinal models allowed insights into directional within-person relations between these symptoms over time. ‘*Child cannot sit still, is restless, or hyperactive*’ was found to have the strongest direct temporal influence on internalising symptoms overall, with higher scores preceding an increase in the anxiety item ‘*Child is too fearful or anxious*’ while some items from the inattentive ADHD domain (‘*Child is distractible, has trouble sticking to any activity’* and ‘*Child cannot concentrate, cannot pay attention for long’*) preceded higher scores on the anxiety item ‘*Child is nervous, high-strung or tense*’. This suggests that, at the within-person level, anxiety symptoms may be exacerbated by ADHD symptoms. Previous research has suggested that the development of co-occurring anxiety could be related to the secondary effects of psychosocial difficulties associated with ADHD such as low educational achievement and social functioning deficits (Galéra et al., 2009). Consistent exposure to such difficulties has wide ranging negative effects and has also been found to increase the risk of developing anxiety problems (Bishop et al., 2019; Mazzone et al., 2007). Another contributing factor to the observed association of restlessness preceding anxiety could be that restlessness is in fact an early sign of anxiety rather than an ADHD symptom per se. Further research is needed to illuminate the reasons for these associations.

Examining the within-person longitudinal effects of internalising symptoms on ADHD symptoms, results suggested that the anxiety item ‘*Child is worried, high-strung or tense*’ showed directional relations with inattentive symptoms. This indicates that internalising symptoms potentially aggravate ADHD symptoms in middle childhood, adding to emerging evidence that ADHD and internalising problems share reciprocal within-person relations (Murray et al., 2020a, b). These findings are also consistent with the hypothesis that one reason why some individuals only develop ADHD symptoms later in life is that these individuals have not been exposed to the same environmental risk load as those who develop symptoms early in life (Lunsford‐Avery & Kollins, 2018). These individuals showing increased ADHD symptom following anxiety symptoms may already be at risk of ADHD symptoms with anxiety acting as a triggering factor. Another potential mechanism underlying the observed temporal association of anxiety symptoms with inattentive symptoms is that anxiety inhibits the appropriate allocation of working memory resources, negatively impacting executive functioning, and in turn leading to increased inattentive behaviour (Eysenck et al., 2007; Zainal & Newman, 2020). Similarly, the observed directional association may capture the fact that worrying interferes with an individual’s concentration, manifesting as inattention symptoms.

Temporal within-person networks also highlighted relations between symptoms of depression and hyperactivity/impulsivity. In particular, over time, ‘*Child is not as happy as other children*’ or ‘*Child appears miserable, distressed, or unhappy*’ was associated with increases in symptoms relating to restlessness and fidgeting. This finding is consistent with the exacerbation hypothesis which proposes that children high on ADHD and internalising symptoms, usually anxiety, display more severe behavioural symptoms due to the combined effect of impairments in inhibitory control associated with both difficulties (Becker et al., 2012). Alternatively, the observed associations could also simply reflect the fact that depression is sometimes accompanied by a state of agitation which manifests in symptoms such as restlessness (Winstanley et al., 2006). Our results further suggest that within-person links between depression items and ADHD are mostly mediated through difficulties in the anxiety domain as, apart from impulsivity showing an association with increased distress, ADHD symptoms only had indirect temporal effects on depression through anxiety symptoms. This is in line with previous research on the relations between depression and ADHD which has found that their relation is often mediated through anxiety and disruptive behaviour disorders (Roy et al., 2014).

Finally, the longitudinal within-person network also highlighted some potentially revealing relations between different ADHD symptoms. In particular, the two impulsivity items, ‘*Child is impulsive, acts without thinking*’ and *‘Child has difficulty awaiting turn in games or groups*’, were only indirectly connected through the item *‘Child is distractible, has trouble sticking to any activity*’. Considering that the GVAR model is based on partial correlations, this indicates that any relation between the two impulsivity items may be better explained by their shared relation with distractibility. Specifically, the directional effects suggest that increased impulsivity may lead to higher distractibility symptoms which might subsequently increase difficulties with awaiting turns. Another reason why the impulsivity items were not directly connected could be that impulsivity is multi-faceted and these two items may capture different forms of impulsivity that are based on distinct underlying deficits. Difficulties awaiting turns is likely closely related to delay aversion which has been associated with temporal processing deficits in ADHD whereas the more general ‘C*hild is impulsive, acts without thinking*’ item might capture more fundamental difficulties with inhibitory control (Winstanley et al., 2006).

Findings of the current study support current clinical best practice. In particular, results highlight the need for screening children who show high ADHD symptomatology also for internalising problems and vice versa, especially if they show symptoms of anxiety and for paying particular attention to the presence of bridge symptoms that may put a child at risk of the development or escalation of co-occurring issues. Interventions such as Cognitive Behavioural Therapy (CBT) may be particularly beneficial for children suffering from both ADHD and internalising symptoms as symptoms such as worrying, which was found to bridge these two mental health domains, have been found to respond very well to CBT (Barrett et al., 2001). Intervention studies have further shown that targeting anxiety symptoms with CBT also leads to a reduction in ADHD symptoms (Gould et al., 2018), thus showing promise for reducing the co-occurrence of ADHD and internalising problems.

### Limitations and Future Directions

The main limitation of this study is that our measure of ADHD and internalising problems relied on relatively few items, allowing only limited inference on bridge symptoms between these areas of psychosocial functioning. Future studies should use a broader range of symptoms and ideally the full set of DSM-5 symptoms for ADHD and internalising disorders to investigate the development of their symptom networks over time. Also, results of the current study are based on a community sample and consequently might not generalise to clinical populations. While using community samples has some important advantages, such as minimising the risk for Berkson’s bias (i.e. the overestimation of symptom co-occurrence; Berkson, 1946), future studies have to be conducted to investigate whether the observed relations would unfold differently for children at the clinical end of the spectrum. Further, the current study relied on estimating associations between individual symptoms measured by single-items, which means that measurement error was likely greater than when using a composite of multiple items. Future research should investigate the reliability of these single-item measurements and ideally use a multi-item measure of individual symptoms. In addition, some methodological considerations need to be addressed in future research. While we were able to show that cross-sectional networks were fairly similar between parent- and teacher-reports, we were not able to replicate the longitudinal symptom network using the teacher-reported data as the high collinearity between some of the SBQ items led to estimation difficulties. Further, the GVAR model was exploratory in nature and will need to be replicated in independent data. The development of confirmatory longitudinal network models will be important to enable testing of the replicability of longitudinal networks because concerns have been raised regarding their stability (Jordan et al., 2020).

In terms of other future directions, it will be valuable to extend the symptom networks to range from childhood to adolescence and into adulthood in order to provide a comprehensive picture of ADHD and internalising symptom relations over the lifespan. In addition, future studies should also include features associated with a broader ADHD phenotype in their analyses. In particular, emotional dysregulation and sluggish cognitive tempo have been identified as potential mediators between symptoms of ADHD and internalising problems (Anastopoulos et al., 2011; Sevincok et al., 2020). Due to sample size constraints, the current study did not investigate whether factors such as child gender, ADHD subtype or symptom severity might lead to symptom networks unfolding differently concurrently and temporally. This would be valuable to explore in future research using methods such as moderated network analyses (Haslbeck et al., 2019). Finally, future research should also investigate whether psychological or pharmacological treatments for ADHD or internalising symptoms have an effect on the overall network structure and especially on edges between ADHD and internalising symptoms. Some evidence from intervention studies suggests that treatment of anxiety and ADHD leads to a greater reduction in symptoms for concurrent anxiety and hyperactive/impulsive symptoms than for concurrent anxiety and inattentive symptoms (Jarrett, 2013). This is in line with results from our cross-sectional networks which suggest that anxiety shares closer links with hyperactivity/impulsivity symptoms than inattentive symptoms. Thus, these preliminary results highlight that investigating the effects of treatments on symptom networks could pave the way for developing interventions that reduce the chances of developing co-occurring symptoms of ADHD or internalising problems by targeting specific bridge symptoms.

## Conclusion

This study offered unique insights into the developmental relations of internalising and ADHD symptoms. Results of cross-sectional and within-person longitudinal analyses highlight that ADHD shares reciprocal relations with internalising symptoms through a number of potential bridge symptoms that are primarily connected to symptoms from the anxiety domain. Future studies are needed to better understand the mechanisms through which ADHD and internalising symptoms affect each other and to evaluate the feasibility of targeting specific bridge symptoms in interventions.

## Supplementary Information

Below is the link to the electronic supplementary material.

Supplementary file1 (CSV 14 KB)

Supplementary file2 (CSV 14 KB)

Supplementary file3 (DOCX 8515 KB)

## Data Availability

Data available upon request.

## Code Availability

Code available upon request.
