# Supplementary material for: Developmental Relations Between Internalising Problems and ADHD in Childhood: a Symptom Level Perspective
Source: Res Child Adolesc Psychopathol. 2021 Aug 7;49(12):1567–79. doi: 10.1007/s10802-021-00856-3 (PMC8557182; doi:10.1007/s10802-021-00856-3)
Supplement: Supplementary file 3 — Supplementary file3 (DOCX 8515 KB) [file 10802_2021_856_MOESM3_ESM.docx]

**Research on Child and Adolescent Psychopathology**

**Supplementary Materials**

**Developmental Relations between Internalising Problems and ADHD in Childhood: A Symptom Level Perspective**

Lydia Gabriela Speyera, Manuel Eisnerb,c, Denis Ribeaudc, Michelle Lucianoa, Bonnie Auyeunga,d, and Aja Louise Murraya,b

**Affiliations:** aDepartment of Psychology, Univsersity of Edinburgh, Edinburgh, United Kingdom; bViolence Research Centre, Institute of Criminology, University of Cambridge, Cambridge, United Kingdom; cJacobs Center for Productive Youth Development, University of Zurich, Zurich, Switzerland; and dAutism Research Centre, Department of Psychiatry, University of Cambridge, Cambridge, United Kingdom;

**Address correspondence to:** Lydia Gabriela Speyer, Department of Psychology, University of Edinburgh, 7 George Square, Edinburgh, EH8 9JZ, United Kingdom, [[lspeyer@ed.ac.uk, 00436506914172].](mailto:lspeyer@exseed.ed.ac.uk)

**
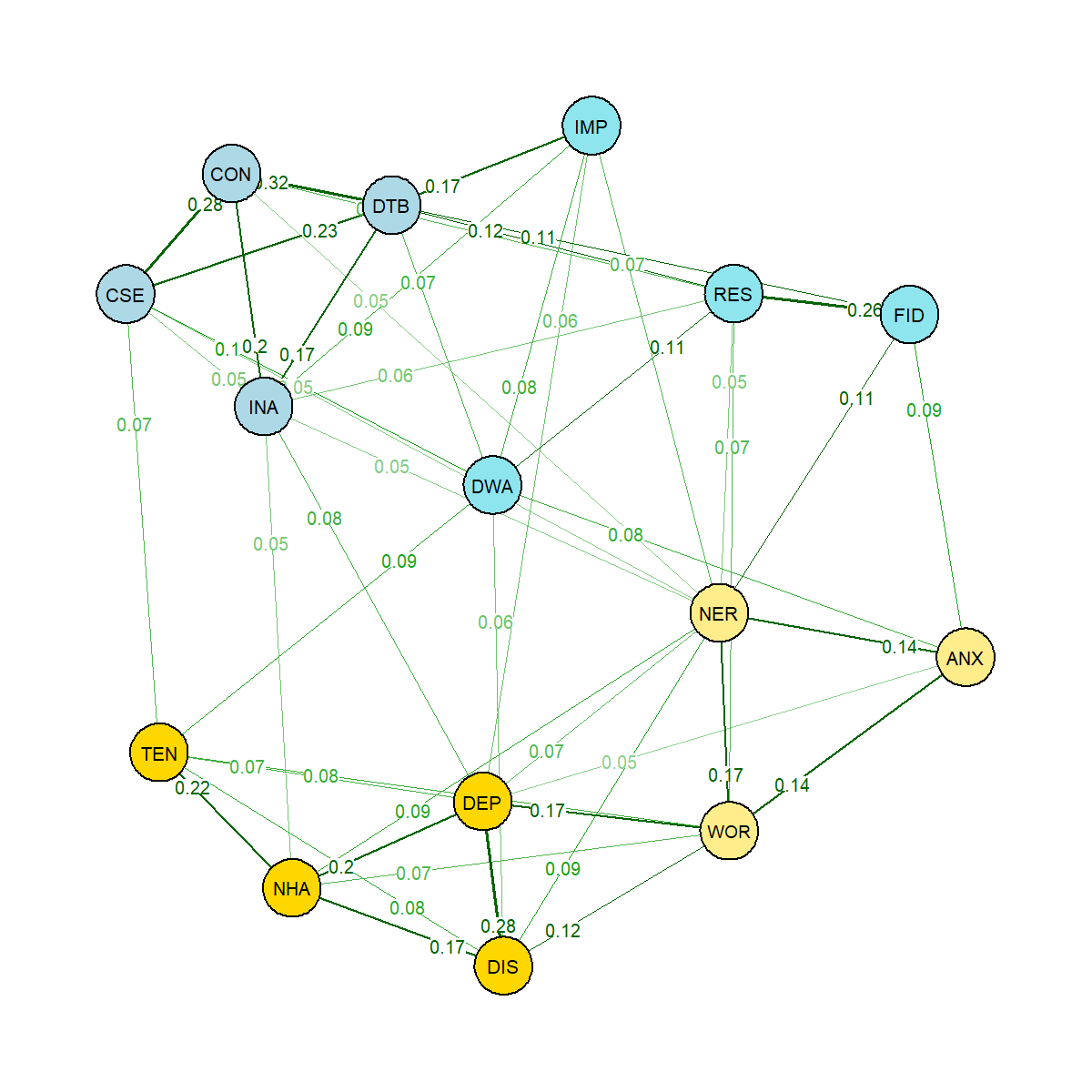
**

**Fig. S1:** Contemporaneous within-person network for parent-reported symptoms standardised to partial correlations. Green edges (solid lines) indicate positive effects. NER: *nervous*, ANX: *anxious*, WOR: *worried*; DEP: *depressed*, NHA: *not as happy as other children*, TEN: *trouble enjoying themselves*, DIS: *distressed*, IMP: *impulsive*, DWA: *difficulty awaiting turns*, RES: *restless*, FID: *fidgets*, CSE: *cannot settle to anything*, DTB: *distractible*, CON: *cannot* *concentrate*, INA: *inattentive*;

**
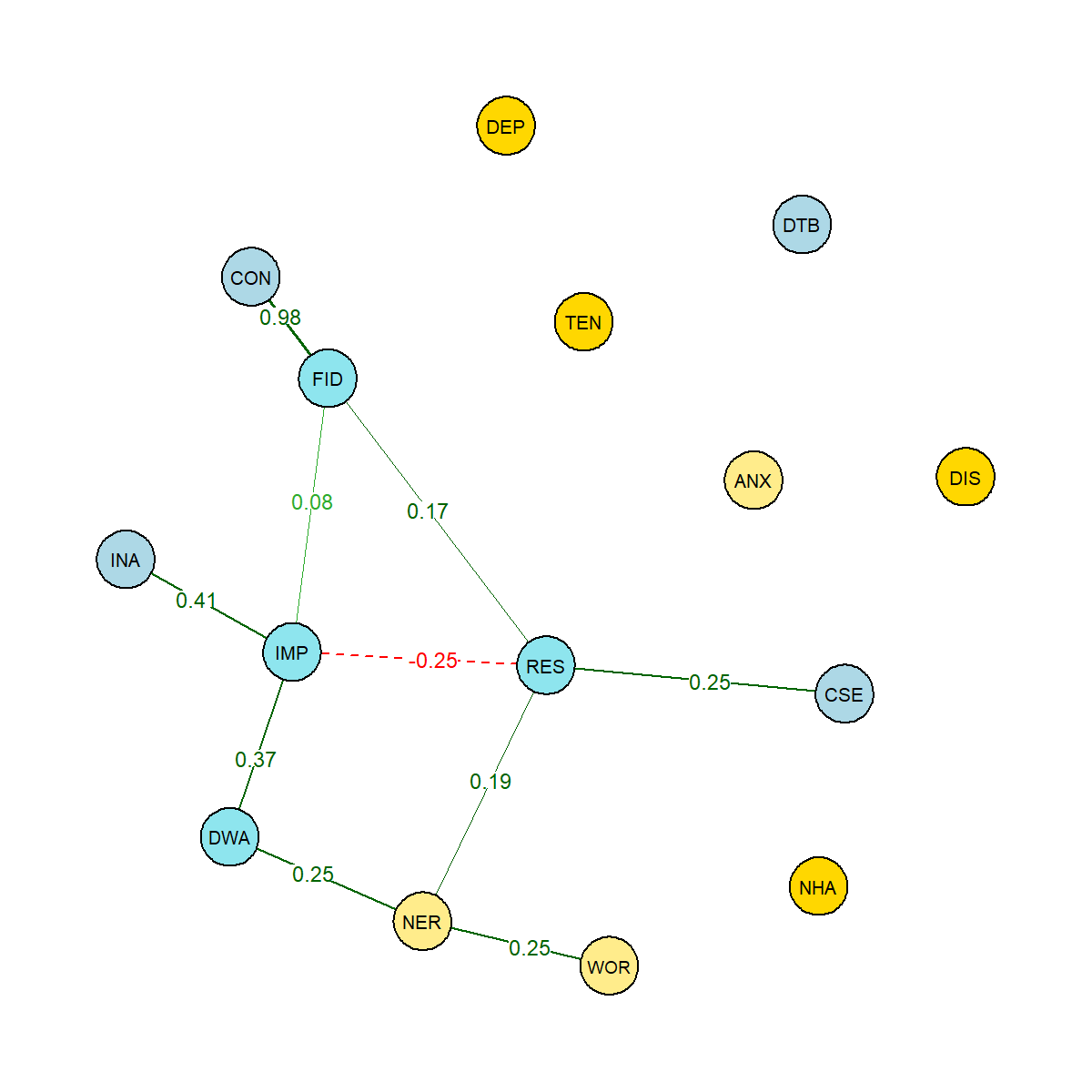
**

**Fig. S2** Between-person network for parent-reported symptoms standardised to partial correlations. Green edges (solid lines) indicate positive effects; red edges (dashed lines) indicate negative effects. NER: *nervous*, ANX: *anxious*, WOR: *worried*; DEP: *depressed*, NHA: *not as happy as other children*, TEN: *trouble enjoying themselves*, DIS: *distressed*, IMP: *impulsive*, DWA: *difficulty awaiting turns*, RES: *restless*, FID: *fidgets*, CSE: *cannot settle to anything*, DTB: *distractible*, CON: *cannot* *concentrate*, INA: *inattentive*;

| **Table S1**: Descriptive Statistics | | | | | | | | |
| --- | --- | --- | --- | --- | --- | --- | --- | --- |
| Variable | *N* | *Mean* | *SD* | *Min* | *Max* | *Range* | *Skew* | *Kurtosis* |
| NER Age 7 - Parent | 1229 | 1.88 | 0.92 | 1 | 5 | 4 | 0.79 | -0.06 |
| ANX Age 7 - Parent | 1228 | 2.07 | 1.11 | 1 | 5 | 4 | 0.72 | -0.41 |
| WOR Age 7 - Parent | 1223 | 1.85 | 0.89 | 1 | 5 | 4 | 0.79 | 0.01 |
| DEP Age 7 - Parent | 1228 | 1.47 | 0.70 | 1 | 5 | 4 | 1.40 | 1.60 |
| NHA Age 7 - Parent | 1213 | 1.30 | 0.64 | 1 | 5 | 4 | 2.31 | 5.29 |
| TEN Age 7 - Parent | 1226 | 1.30 | 0.65 | 1 | 5 | 4 | 2.61 | 7.87 |
| DIS Age 7 - Parent | 1223 | 1.44 | 0.71 | 1 | 5 | 4 | 1.57 | 2.08 |
| IMP Age 7 - Parent | 1219 | 2.27 | 1.02 | 1 | 5 | 4 | 0.41 | -0.45 |
| DWA Age 7 - Parent | 1219 | 2.46 | 1.09 | 1 | 5 | 4 | 0.19 | -0.72 |
| RES Age 7 - Parent | 1226 | 2.22 | 1.18 | 1 | 5 | 4 | 0.65 | -0.53 |
| FID Age 7 - Parent | 1224 | 2.39 | 1.24 | 1 | 5 | 4 | 0.45 | -0.81 |
| CSE Age 7 - Parent | 1220 | 1.96 | 0.97 | 1 | 5 | 4 | 0.74 | -0.16 |
| DTB Age 7 - Parent | 1224 | 2.31 | 1.03 | 1 | 5 | 4 | 0.30 | -0.58 |
| CON Age 7 - Parent | 1223 | 2.00 | 0.97 | 1 | 5 | 4 | 0.69 | -0.12 |
| INA Age 7 - Parent | 1225 | 1.96 | 0.92 | 1 | 5 | 4 | 0.56 | -0.43 |
| NER Age 9 - Parent | 1179 | 2.05 | 0.92 | 1 | 5 | 4 | 0.54 | -0.26 |
| ANX Age 9 - Parent | 1180 | 2.13 | 1.04 | 1 | 5 | 4 | 0.70 | -0.12 |
| WOR Age 9 - Parent | 1178 | 2.00 | 0.86 | 1 | 5 | 4 | 0.45 | -0.33 |
| DEP Age 9 - Parent | 1180 | 1.69 | 0.74 | 1 | 5 | 4 | 0.74 | -0.21 |
| NHA Age 9 - Parent | 1169 | 1.50 | 0.74 | 1 | 5 | 4 | 1.37 | 1.29 |
| TEN Age 9 - Parent | 1178 | 1.47 | 0.74 | 1 | 5 | 4 | 1.65 | 2.56 |
| DIS Age 9 - Parent | 1178 | 1.61 | 0.73 | 1 | 5 | 4 | 0.94 | 0.24 |
| IMP Age 9 - Parent | 1176 | 2.30 | 0.97 | 1 | 5 | 4 | 0.41 | -0.15 |
| DWA Age 9 - Parent | 1164 | 2.39 | 1.05 | 1 | 5 | 4 | 0.29 | -0.60 |
| RES Age 9 - Parent | 1179 | 2.18 | 1.10 | 1 | 5 | 4 | 0.65 | -0.39 |
| FID Age 9 - Parent | 1176 | 2.37 | 1.16 | 1 | 5 | 4 | 0.48 | -0.63 |
| CSE Age 9 - Parent | 1175 | 2.19 | 0.96 | 1 | 5 | 4 | 0.48 | -0.30 |
| DTB Age 9 - Parent | 1179 | 2.48 | 1.03 | 1 | 5 | 4 | 0.34 | -0.36 |
| CON Age 9 - Parent | 1178 | 2.25 | 0.97 | 1 | 5 | 4 | 0.51 | -0.11 |
| INA Age 9 - Parent | 1179 | 2.15 | 0.94 | 1 | 5 | 4 | 0.38 | -0.50 |
| NER Age 11 - Parent | 1072 | 2.09 | 0.90 | 1 | 5 | 4 | 0.53 | -0.10 |
| ANX Age 11 - Parent | 1071 | 2.10 | 1.05 | 1 | 5 | 4 | 0.70 | -0.26 |
| WOR Age 11 - Parent | 1072 | 2.12 | 0.88 | 1 | 5 | 4 | 0.39 | -0.40 |
| DEP Age 11 - Parent | 1071 | 1.75 | 0.77 | 1 | 5 | 4 | 0.79 | 0.26 |
| NHA Age 11 - Parent | 1071 | 1.64 | 0.83 | 1 | 5 | 4 | 1.25 | 1.20 |
| TEN Age 11 - Parent | 1072 | 1.54 | 0.74 | 1 | 5 | 4 | 1.32 | 1.64 |
| DIS Age 11 - Parent | 1071 | 1.67 | 0.77 | 1 | 5 | 4 | 0.96 | 0.47 |
| IMP Age 11 - Parent | 1070 | 2.27 | 0.96 | 1 | 5 | 4 | 0.47 | -0.14 |
| DWA Age 11 - Parent | 1069 | 2.26 | 0.99 | 1 | 5 | 4 | 0.44 | -0.39 |
| RES Age 11 - Parent | 1072 | 2.07 | 1.11 | 1 | 5 | 4 | 0.78 | -0.25 |
| FID Age 11 - Parent | 1073 | 2.36 | 1.19 | 1 | 5 | 4 | 0.53 | -0.64 |
| CSE Age 11 - Parent | 1071 | 2.13 | 0.94 | 1 | 5 | 4 | 0.53 | -0.19 |
| DTB Age 11 - Parent | 1068 | 2.44 | 1.06 | 1 | 5 | 4 | 0.35 | -0.51 |
| CON Age 11 - Parent | 1072 | 2.29 | 1.00 | 1 | 5 | 4 | 0.50 | -0.26 |
| INA Age 11 - Parent | 1072 | 2.24 | 0.92 | 1 | 5 | 4 | 0.38 | -0.17 |
| NER Age 7 - Teacher | 1343 | 2.24 | 1.10 | 1 | 5 | 4 | 0.54 | -0.57 |
| ANX Age 7 - Teacher | 1343 | 2.02 | 1.04 | 1 | 5 | 4 | 0.82 | -0.04 |
| WOR Age 7 - Teacher | 1336 | 2.05 | 0.99 | 1 | 5 | 4 | 0.66 | -0.27 |
| DEP Age 7 - Teacher | 1340 | 1.79 | 0.93 | 1 | 5 | 4 | 1.07 | 0.62 |
| NHA Age 7 - Teacher | 1333 | 1.77 | 0.99 | 1 | 5 | 4 | 1.18 | 0.61 |
| TEN Age 7 - Teacher | 1337 | 1.68 | 0.87 | 1 | 5 | 4 | 1.24 | 1.07 |
| DIS Age 7 - Teacher | 1343 | 1.54 | 0.86 | 1 | 5 | 4 | 1.70 | 2.62 |
| IMP Age 7 - Teacher | 1342 | 2.24 | 1.17 | 1 | 5 | 4 | 0.65 | -0.46 |
| DWA Age 7 - Teacher | 1339 | 2.28 | 1.23 | 1 | 5 | 4 | 0.64 | -0.61 |
| RES Age 7 - Teacher | 1346 | 2.11 | 1.20 | 1 | 5 | 4 | 0.82 | -0.37 |
| FID Age 7 - Teacher | 1339 | 1.99 | 1.17 | 1 | 5 | 4 | 1.03 | 0.09 |
| CSE Age 7 - Teacher | 1344 | 2.23 | 1.14 | 1 | 5 | 4 | 0.63 | -0.50 |
| DTB Age 7 - Teacher | 1342 | 2.50 | 1.24 | 1 | 5 | 4 | 0.42 | -0.83 |
| CON Age 7 - Teacher | 1342 | 2.35 | 1.17 | 1 | 5 | 4 | 0.53 | -0.62 |
| INA Age 7 - Teacher | 1331 | 2.28 | 1.11 | 1 | 5 | 4 | 0.58 | -0.43 |
| NER Age 9 - Teacher | 1291 | 2.19 | 1.01 | 1 | 5 | 4 | 0.50 | -0.39 |
| ANX Age 9 - Teacher | 1291 | 1.92 | 0.94 | 1 | 5 | 4 | 0.83 | 0.11 |
| WOR Age 9 - Teacher | 1291 | 2.02 | 0.94 | 1 | 5 | 4 | 0.65 | -0.12 |
| DEP Age 9 - Teacher | 1291 | 1.79 | 0.90 | 1 | 5 | 4 | 1.06 | 0.69 |
| NHA Age 9 - Teacher | 1288 | 1.77 | 0.93 | 1 | 5 | 4 | 1.11 | 0.65 |
| TEN Age 9 - Teacher | 1289 | 1.63 | 0.83 | 1 | 5 | 4 | 1.17 | 0.78 |
| DIS Age 9 - Teacher | 1289 | 1.56 | 0.81 | 1 | 5 | 4 | 1.48 | 1.96 |
| IMP Age 9 - Teacher | 1291 | 2.07 | 1.11 | 1 | 5 | 4 | 0.86 | 0.01 |
| DWA Age 9 - Teacher | 1291 | 2.05 | 1.15 | 1 | 5 | 4 | 0.92 | -0.03 |
| RES Age 9 - Teacher | 1292 | 1.92 | 1.12 | 1 | 5 | 4 | 1.13 | 0.46 |
| FID Age 9 - Teacher | 1290 | 1.83 | 1.11 | 1 | 5 | 4 | 1.28 | 0.76 |
| CSE Age 9 - Teacher | 1292 | 2.05 | 1.07 | 1 | 5 | 4 | 0.81 | -0.09 |
| DTB Age 9 - Teacher | 1292 | 2.28 | 1.17 | 1 | 5 | 4 | 0.60 | -0.56 |
| CON Age 9 - Teacher | 1293 | 2.18 | 1.14 | 1 | 5 | 4 | 0.75 | -0.24 |
| INA Age 9 - Teacher | 1291 | 2.16 | 1.10 | 1 | 5 | 4 | 0.72 | -0.17 |
| NER Age 11 - Teacher | 1062 | 2.24 | 1.01 | 1 | 5 | 4 | 0.49 | -0.33 |
| ANX Age 11 - Teacher | 1060 | 1.85 | 0.94 | 1 | 5 | 4 | 1.05 | 0.73 |
| WOR Age 11 - Teacher | 1063 | 2.06 | 0.93 | 1 | 5 | 4 | 0.67 | 0.04 |
| DEP Age 11 - Teacher | 1063 | 1.89 | 0.96 | 1 | 5 | 4 | 0.91 | 0.21 |
| NHA Age 11 - Teacher | 1042 | 1.88 | 0.99 | 1 | 5 | 4 | 0.92 | 0.05 |
| TEN Age 11 - Teacher | 1045 | 1.75 | 0.92 | 1 | 5 | 4 | 1.16 | 0.90 |
| DIS Age 11 - Teacher | 1063 | 1.63 | 0.86 | 1 | 5 | 4 | 1.34 | 1.32 |
| IMP Age 11 - Teacher | 1059 | 2.08 | 1.13 | 1 | 5 | 4 | 0.85 | -0.07 |
| DWA Age 11 - Teacher | 1063 | 1.93 | 1.10 | 1 | 5 | 4 | 1.00 | 0.04 |
| RES Age 11 - Teacher | 1061 | 1.86 | 1.13 | 1 | 5 | 4 | 1.23 | 0.59 |
| FID Age 11 - Teacher | 1062 | 1.75 | 1.11 | 1 | 5 | 4 | 1.44 | 1.15 |
| CSE Age 11 - Teacher | 1063 | 2.09 | 1.17 | 1 | 5 | 4 | 0.80 | -0.37 |
| DTB Age 11 - Teacher | 1062 | 2.39 | 1.26 | 1 | 5 | 4 | 0.51 | -0.85 |
| CON Age 11 - Teacher | 1060 | 2.23 | 1.20 | 1 | 5 | 4 | 0.67 | -0.53 |
| INA Age 11 - Teacher | 1062 | 2.25 | 1.13 | 1 | 5 | 4 | 0.59 | -0.50 |
| *Note.* NER: *nervous*, ANX: *anxious*, WOR: *worried*; DEP: *depressed*, NHA: *not as happy as other children*, TEN: *trouble enjoying themselves*, DIS: *distressed*, IMP: *impulsive*, DWA: *difficulty awaiting turns*, RES: *restless*, FID: *fidgets*, CSE: *cannot settle to anything*, DTB: *distractible*, CON: *cannot* *concentrate*, INA: *inattentive*; | | | | | | | | |

| **Table S2:** Bootstrapped Confidence Intervals for Cross-Sectional Networks Age 7 | | | | | | | | | |
| --- | --- | --- | --- | --- | --- | --- | --- | --- | --- |
| **Parent-Report Age 7** | | | | | **Teacher-Report Age 7** | | | | |
| *Edge* | *Weight* | *Mean* | *CI_lower_* | *CI_upper_* | *Edge* | *Weight* | *Mean* | *CI_lower_* | *CI_upper_* |
| 02 - 03 | 0.12 | 0.08 | -0.02 | 0.26 | 02 - 03 | 0.17 | 0.17 | 0.12 | 0.23 |
| 02 - 04 | 0.21 | 0.21 | 0.15 | 0.28 | 02 - 04 | 0.27 | 0.27 | 0.21 | 0.32 |
| 02 - 12 | 0.11 | 0.10 | 0.00 | 0.22 | 02 - 05 | 0.06 | 0.05 | -0.02 | 0.14 |
| 02 - 13 | 0.09 | 0.05 | -0.03 | 0.21 | 02 - 10 | 0.11 | 0.11 | 0.05 | 0.17 |
| 03 - 04 | 0.13 | 0.11 | 0.01 | 0.26 | 02 - 11 | 0.06 | 0.05 | -0.03 | 0.15 |
| 04 - 05 | 0.17 | 0.16 | 0.10 | 0.24 | 02 - 12 | 0.05 | 0.04 | -0.02 | 0.12 |
| 04 - 08 | 0.13 | 0.11 | 0.01 | 0.25 | 02 - 13 | 0.11 | 0.10 | 0.05 | 0.16 |
| 05 - 06 | 0.22 | 0.22 | 0.15 | 0.29 | 03 - 04 | 0.42 | 0.42 | 0.36 | 0.47 |
| 05 - 07 | 0.14 | 0.14 | 0.04 | 0.25 | 03 - 07 | 0.09 | 0.06 | -0.01 | 0.18 |
| 05 - 08 | 0.20 | 0.20 | 0.14 | 0.27 | 04 - 05 | 0.21 | 0.21 | 0.16 | 0.27 |
| 06 - 07 | 0.23 | 0.22 | 0.15 | 0.30 | 04 - 06 | 0.07 | 0.05 | -0.01 | 0.14 |
| 06 - 08 | 0.13 | 0.12 | 0.02 | 0.25 | 04 - 08 | 0.06 | 0.05 | -0.02 | 0.14 |
| 07 - 08 | 0.12 | 0.10 | -0.01 | 0.26 | 05 - 06 | 0.37 | 0.37 | 0.31 | 0.43 |
| 10 - 11 | 0.19 | 0.19 | 0.13 | 0.25 | 05 - 07 | 0.18 | 0.17 | 0.11 | 0.24 |
| 10 - 17 | 0.20 | 0.20 | 0.14 | 0.26 | 05 - 08 | 0.28 | 0.28 | 0.22 | 0.34 |
| 12 - 13 | 0.48 | 0.48 | 0.43 | 0.53 | 06 - 07 | 0.29 | 0.29 | 0.23 | 0.36 |
| 12 – 15 | 0.09 | 0.06 | -0.02 | 0.20 | 06 - 08 | 0.30 | 0.30 | 0.23 | 0.37 |
| 12 - 16 | 0.08 | 0.05 | -0.03 | 0.18 | 06 - 17 | 0.05 | 0.03 | -0.01 | 0.11 |
| 14 - 15 | 0.20 | 0.20 | 0.14 | 0.26 | 07 - 08 | 0.13 | 0.13 | 0.06 | 0.20 |
| 14 - 16 | 0.27 | 0.27 | 0.21 | 0.33 | 10 - 11 | 0.40 | 0.39 | 0.34 | 0.46 |
| 15 - 16 | 0.31 | 0.31 | 0.25 | 0.37 | 10 - 12 | 0.18 | 0.17 | 0.13 | 0.23 |
| 15 - 17 | 0.17 | 0.17 | 0.11 | 0.23 | 10 - 17 | 0.15 | 0.15 | 0.11 | 0.20 |
| 16 - 17 | 0.24 | 0.24 | 0.18 | 0.30 | 11 - 12 | 0.24 | 0.24 | 0.19 | 0.30 |
|  |  |  |  |  | 11 - 13 | 0.08 | 0.07 | 0.01 | 0.14 |
|  |  |  |  |  | 12 - 13 | 0.65 | 0.65 | 0.61 | 0.70 |
|  |  |  |  |  | 12 - 14 | 0.07 | 0.07 | 0.02 | 0.13 |
|  |  |  |  |  | 12 - 15 | 0.06 | 0.05 | 0.02 | 0.10 |
|  |  |  |  |  | 13 - 14 | 0.05 | 0.06 | -0.01 | 0.12 |
|  |  |  |  |  | 13 - 16 | 0.03 | 0.03 | -0.01 | 0.08 |
|  |  |  |  |  | 14 - 15 | 0.28 | 0.28 | 0.21 | 0.34 |
|  |  |  |  |  | 14 - 16 | 0.29 | 0.29 | 0.23 | 0.35 |
|  |  |  |  |  | 14 - 17 | 0.04 | 0.04 | -0.03 | 0.11 |
|  |  |  |  |  | 15 - 16 | 0.52 | 0.51 | 0.46 | 0.58 |
|  |  |  |  |  | 15 - 17 | 0.13 | 0.13 | 0.06 | 0.19 |
|  |  |  |  |  | 16 - 17 | 0.40 | 0.40 | 0.35 | 0.46 |
| *Note.* Weight: edge weight in the form of partial correlations; Mean: bootstrapped mean edge weight; CI: 95% confidence intervals | | | | | | | | | |

| **Table S3**: Bootstrapped Confidence Intervals for Cross-Sectional Networks Age 9 | | | | | | | | | |
| --- | --- | --- | --- | --- | --- | --- | --- | --- | --- |
| **Parent-Report Age 9** | | | | | **Teacher-Report Age 9** | | | | |
| *Edge* | *Weight* | *Mean* | *CI_lower_* | *CI_upper_* | *Edge* | *Weight* | *Mean* | *CI_lower_* | *CI_upper_* |
| 02 - 03 | 0.14 | 0.12 | 0.02 | 0.26 | 02 - 03 | 0.16 | 0.16 | 0.10 | 0.22 |
| 02 - 04 | 0.20 | 0.20 | 0.14 | 0.27 | 02 - 04 | 0.31 | 0.32 | 0.26 | 0.37 |
| 02 - 08 | 0.10 | 0.07 | -0.02 | 0.22 | 02 - 10 | 0.10 | 0.09 | 0.02 | 0.17 |
| 03 - 04 | 0.16 | 0.15 | 0.07 | 0.25 | 02 - 11 | 0.10 | 0.11 | 0.03 | 0.17 |
| 04 - 05 | 0.18 | 0.18 | 0.12 | 0.24 | 02 - 12 | 0.04 | 0.02 | -0.02 | 0.10 |
| 04 - 08 | 0.13 | 0.12 | 0.04 | 0.22 | 02 - 13 | 0.08 | 0.09 | 0.03 | 0.13 |
| 05 - 06 | 0.10 | 0.07 | -0.02 | 0.22 | 03 - 04 | 0.42 | 0.42 | 0.37 | 0.48 |
| 05 - 08 | 0.24 | 0.23 | 0.18 | 0.29 | 03 - 05 | 0.09 | 0.08 | 0.02 | 0.16 |
| 06 - 07 | 0.29 | 0.29 | 0.22 | 0.35 | 04 - 05 | 0.22 | 0.23 | 0.17 | 0.28 |
| 06 - 08 | 0.27 | 0.27 | 0.20 | 0.33 | 04 - 06 | 0.10 | 0.09 | 0.04 | 0.15 |
| 10 - 11 | 0.17 | 0.17 | 0.10 | 0.24 | 05 - 06 | 0.43 | 0.43 | 0.37 | 0.49 |
| 10 - 13 | 0.11 | 0.07 | -0.02 | 0.24 | 05 - 07 | 0.08 | 0.07 | 0.00 | 0.16 |
| 10 - 17 | 0.13 | 0.13 | 0.04 | 0.22 | 05 - 08 | 0.23 | 0.23 | 0.17 | 0.30 |
| 11 – 12 | 0.11 | 0.09 | -0.01 | 0.23 | 06 - 07 | 0.34 | 0.34 | 0.27 | 0.40 |
| 12 - 13 | 0.12 | 0.10 | 0.00 | 0.24 | 06 - 08 | 0.26 | 0.26 | 0.20 | 0.33 |
| 12 - 14 | 0.46 | 0.45 | 0.40 | 0.51 | 07 - 08 | 0.19 | 0.19 | 0.12 | 0.25 |
| 12 - 16 | 0.10 | 0.09 | 0.00 | 0.20 | 10 - 11 | 0.41 | 0.41 | 0.35 | 0.47 |
| 13-15 | 0.08 | 0.06 | -0.02 | 0.18 | 10 - 12 | 0.11 | 0.11 | 0.05 | 0.17 |
| 14 - 15 | 0.10 | 0.09 | 0.01 | 0.19 | 10 - 15 | 0.06 | 0.05 | 0.00 | 0.11 |
| 14 - 16 | 0.22 | 0.21 | 0.15 | 0.28 | 10 - 17 | 0.08 | 0.08 | 0.02 | 0.15 |
| 15 - 16 | 0.28 | 0.28 | 0.22 | 0.35 | 11 - 12 | 0.27 | 0.26 | 0.21 | 0.32 |
| 15 - 17 | 0.40 | 0.40 | 0.34 | 0.46 | 12 - 13 | 0.69 | 0.69 | 0.64 | 0.73 |
| 16 - 17 | 0.16 | 0.16 | 0.10 | 0.22 | 12 - 14 | 0.08 | 0.08 | 0.03 | 0.14 |
|  |  |  |  |  | 12 - 15 | 0.03 | 0.02 | -0.01 | 0.07 |
|  |  |  |  |  | 13 - 14 | 0.08 | 0.08 | 0.02 | 0.14 |
|  |  |  |  |  | 14 - 15 | 0.36 | 0.36 | 0.29 | 0.42 |
|  |  |  |  |  | 14 - 16 | 0.24 | 0.23 | 0.17 | 0.31 |
|  |  |  |  |  | 14 - 17 | 0.14 | 0.14 | 0.08 | 0.21 |
|  |  |  |  |  | 15 - 16 | 0.49 | 0.50 | 0.43 | 0.55 |
|  |  |  |  |  | 15 - 17 | 0.13 | 0.13 | 0.06 | 0.20 |
|  |  |  |  |  | 16 - 17 | 0.34 | 0.34 | 0.27 | 0.40 |
| *Note.* Weight: edge weight in the form of partial correlations; Mean: bootstrapped mean edge weight; CI: 95% confidence intervals | | | | | | | | | |

| **Table S4:** Bootstrapped Confidence Intervals for Cross-Sectional Networks Age 11 | | | | | | | | | |
| --- | --- | --- | --- | --- | --- | --- | --- | --- | --- |
| **Parent-Report Age 11** | | | | | **Teacher-Report Age 11** | | | | |
| *Edge* | *Weight* | *Mean* | *CI_lower_* | *CI_upper_* | *Edge* | *Weight* | *Mean* | *CI_lower_* | *CI_upper_* |
| 02 - 03 | 0.12 | 0.09 | -0.02 | 0.25 | 02 - 03 | 0.16 | 0.17 | 0.10 | 0.23 |
| 02 - 04 | 0.19 | 0.19 | 0.12 | 0.26 | 02 - 04 | 0.31 | 0.30 | 0.25 | 0.37 |
| 02 - 08 | 0.11 | 0.10 | 0.01 | 0.22 | 02 - 10 | 0.14 | 0.14 | 0.08 | 0.20 |
| 02 - 13 | 0.13 | 0.12 | 0.02 | 0.23 | 02 - 12 | 0.09 | 0.09 | 0.03 | 0.16 |
| 03 - 04 | 0.15 | 0.15 | 0.05 | 0.26 | 03 - 04 | 0.35 | 0.35 | 0.29 | 0.42 |
| 04 - 05 | 0.19 | 0.19 | 0.12 | 0.25 | 03 - 07 | 0.09 | 0.06 | -0.02 | 0.19 |
| 04 - 08 | 0.11 | 0.09 | -0.01 | 0.22 | 04 - 05 | 0.28 | 0.27 | 0.21 | 0.34 |
| 05 - 06 | 0.22 | 0.22 | 0.15 | 0.28 | 04 - 06 | 0.07 | 0.06 | -0.01 | 0.14 |
| 05 - 08 | 0.33 | 0.33 | 0.27 | 0.40 | 05 - 06 | 0.43 | 0.43 | 0.37 | 0.50 |
| 06 - 07 | 0.18 | 0.18 | 0.12 | 0.25 | 05 - 08 | 0.31 | 0.30 | 0.24 | 0.37 |
| 06 - 08 | 0.23 | 0.23 | 0.16 | 0.29 | 05 - 14 | 0.04 | 0.03 | -0.02 | 0.09 |
| 07 - 08 | 0.12 | 0.10 | 0.00 | 0.24 | 06 - 07 | 0.40 | 0.40 | 0.33 | 0.47 |
| 10 - 11 | 0.14 | 0.13 | 0.03 | 0.26 | 06 - 08 | 0.15 | 0.15 | 0.08 | 0.23 |
| 10 - 13 | 0.12 | 0.10 | 0.01 | 0.24 | 07 - 08 | 0.25 | 0.24 | 0.17 | 0.32 |
| 10 - 15 | 0.10 | 0.08 | -0.01 | 0.20 | 08 - 17 | 0.05 | 0.05 | -0.02 | 0.12 |
| 12 - 13 | 0.13 | 0.12 | 0.03 | 0.23 | 10 - 11 | 0.44 | 0.44 | 0.38 | 0.51 |
| 12 - 15 | 0.12 | 0.10 | 0.00 | 0.23 | 10 - 12 | 0.09 | 0.09 | 0.02 | 0.17 |
| 12 - 16 | 0.45 | 0.44 | 0.39 | 0.50 | 10 - 14 | 0.07 | 0.06 | 0.00 | 0.14 |
| 14 - 15 | 0.09 | 0.08 | 0.00 | 0.19 | 10 – 15 | 0.04 | 0.03 | -0.02 | 0.10 |
| 14 - 16 | 0.11 | 0.10 | 0.03 | 0.19 | 10 - 17 | 0.07 | 0.06 | -0.01 | 0.14 |
| 15 - 16 | 0.27 | 0.27 | 0.20 | 0.34 | 11 - 12 | 0.24 | 0.24 | 0.16 | 0.31 |
| 15 - 17 | 0.32 | 0.32 | 0.25 | 0.38 | 11 - 13 | 0.09 | 0.08 | 0.00 | 0.18 |
| 16 - 17 | 0.34 | 0.34 | 0.28 | 0.41 | 12 - 13 | 0.63 | 0.63 | 0.58 | 0.68 |
|  |  |  |  |  | 12 - 14 | 0.10 | 0.09 | 0.03 | 0.16 |
|  |  |  |  |  | 12 - 15 | 0.05 | 0.04 | -0.01 | 0.11 |
|  |  |  |  |  | 13 - 14 | 0.06 | 0.05 | -0.02 | 0.13 |
|  |  |  |  |  | 14 - 15 | 0.29 | 0.29 | 0.22 | 0.37 |
|  |  |  |  |  | 14 - 16 | 0.32 | 0.32 | 0.24 | 0.39 |
|  |  |  |  |  | 14 - 17 | 0.06 | 0.06 | -0.01 | 0.14 |
|  |  |  |  |  | 15 - 16 | 0.44 | 0.44 | 0.36 | 0.51 |
|  |  |  |  |  | 15 - 17 | 0.21 | 0.21 | 0.14 | 0.29 |
|  |  |  |  |  | 16 - 17 | 0.38 | 0.38 | 0.31 | 0.45 |
| *Note.* Weight: edge weight in the form of partial correlations; Mean: bootstrapped mean edge weight; CI: 95% confidence intervals | | | | | | | | | |

**Supplementary Correlation Tables**

See excel File E1 for correlations for all parent-reported SBQ items and excel File E2 for teacher-reported SBQ items. Lower tringle includes correlations; upper triangle includes significance values.
